# The awareness of enhanced recovery after surgery (ERAS) cesarean delivery guidelines among anesthesiology and reanimation assistants in Turkey; a questionnaire study

**DOI:** 10.1186/s12871-024-02611-9

**Published:** 2024-08-02

**Authors:** Zeliha Dedebagı, Eyyüp Sabri Özden, Mustafa Soner Özcan, Filiz Alkaya Solmaz, Pakize Kırdemir

**Affiliations:** 1 Department of Anesthesiology and Reanimation, Faculty of Medicine, Suleyman Demirel University, Isparta, Turkey; 2 Faculty of Medicine Hospital, Department of Anesthesiology and Reanimation, Suleyman Demirel University, Operating room, Floor:1, Cunur, Isparta, 32000 Turkey

**Keywords:** Anesthesiology, Assistants, Cesarean, Delivery, Enhanced recovery after surgery

## Abstract

**Background:**

To reduce maternal-fetal morbidity and mortality, it is becoming increasingly important for anesthetists to understand and implement enhanced recovery after surgery (ERAS) cesarean delivery guidelines. Our aim was to reveal the knowledge of anesthesia assistants in Turkey about ERAS during cesarean delivery and to increase their awareness of ERAS.

**Methods:**

This descriptive study was conducted in the city of Isparta, Turkey in 2023. The survey, which was approved by the ethics committee, was distributed to participants across Turkey via e-mail and online messages. The survey comprises of a total of 42 questions evaluating perioperative ERAS recommendations.

**Results:**

Of the 404 participants in our survey, 59.9% were associated with university hospitals and 65.8% had completed three or more years of education. A total of 87.9% of the participants were familiar with ERAS; however, only 42.8% had received ERAS training. Although 93.8% of the participants’ institutions performed a cesarean delivery, ERAS recommendations were only implemented at a rate of 48%. This may be due to the absence of an ERAS team, which was identified in our survey at a high rate of 66.6%.

**Conclusion:**

Awareness about ERAS was high among the participants, but the implementation rates of some recommendations were low. The reason for this may be the inability to form a multidisciplinary team and inadequate training of participants. For this purpose, we recommend the formation of a multidisciplinary team for ERAS protocol implementation and increased participant training opportunities.

## Background

Cesarean delivery is the most commonly performed obstetric surgery in the world, and cesarean delivery rates are increasing both globally and in Turkey. The cesarean delivery rate per 1,000 live births in Turkey is 584.20 among the Organization for Economic Cooperation and Development (OECD) countries between 2019 and 2021 [1]. The rising number of cesarean deliveries annually increases the need for perioperative care.

The Enhanced Recovery After Surgery (ERAS) - Cesarean Delivery Guide (CDG) presents evidence-based information from cesarean delivery research in a 3-part guide for the preoperative, intraoperative, and postoperative periods. Its aims to improve maternal and fetal outcomes by increasing the quality and safety of delivery after cesarean delivery [2–4].

The ERAS-CDG for both planned and unplanned cesarean deliveries covers the period between the decision to undergo surgery and discharge from the hospital. The guidelines aim to reduce the metabolic stress associated with surgical trauma, maintain physiological functioning to enable a prompt return to normal activity, and minimize postdischarge pain, complications, and systemic dysfunction [5].

Due to the increasing rates of cesarean deliveries, anesthesiologists are frequently involved in these procedures. As a result, it is crucial for anesthesiologists to be familiar with and implement the ERAS-CDG to decrease maternal-fetal morbidity and mortality, hospitalization, and costs [6, 7].

Our aim was to reveal the knowledge of anesthesia assistants in Turkey about ERAS during cesarean delivery and to increase their awareness of ERAS.

## Methods

This study was approved by the Süleyman Demirel University Clinical Research Ethics Committee (decision number 384,014 dated 10.10.2022). Our online survey study was conducted between November 2022 and March 2023 with the support of the Turkish Society of Anesthesiology and Reanimation by reaching anesthesiology and reanimation assistants in Turkey. This is a descriptive research study. The exclusion criteria were as follows: unwillingness to participate in the research, incomplete data entry, and not being an assistant in anesthesiology and reanimation in Turkey.

The questions in our survey were prepared not with the aim of deterring participants, but by taking into account the recommendations in the direct guide. It was prepared to inform participants about the recommendations in the guide, to explain the recommendations and to increase their awareness. Anesthesiology assistants in Turkey were surveyed regarding their sex, educational level, place of employment, exposure to cesarean delivery in their workplace, utilization of ERAS protocols for cesarean deliveries, ERAS training status, and the existence of an ERAS team. Furthermore, inquiries were made regarding preoperative details, antacid consumption, bowel preparation, sedation, preoperative nourishment, maternal obesity, maternal hypertension, gestational diabetes, anemia, smoking, and antibiotic use following the ERAS-CDG suggestions. Enquiries were made regarding the selection of intraoperative anesthesia, how to prevent anesthesia-induced hypotension, methods of protecting against hypothermia, maintaining euvolemia and recommendations for neonates. The assessment included inquiries on postoperative oral intake, nausea and vomiting, blood glucose management, thromboembolism (TE) precautions, catheter extraction and pain relief. Prior to the survey, an information letter was written and informed consent to participate was obtained from all of the participants in the study. It was stated that the questionnaire was not an examination and that participation in the survey would be voluntary. The 42-question survey form created with Google forms was sent to the participants online via e-mail and message.

### Statistical analysis

The data were transferred to the SPSS 23 (IBM, Inc., Chicago, IL, USA) program and evaluated via statistical analyses. Before conducting any statistical analyses, a thorough check was performed to ensure the absence of data entry errors and to verify that the parameter values were within the expected range. In instances where participants do not respond or forget to respond, the system prevents them from advancing to the subsequent page. The sample size of the study was calculated with the sample formula with a known population. In the sample of the study, 2842 anesthesiology and reanimation assistants in Turkey were included by selecting the purposive sampling method. The sample size of the study was calculated with the sample formula with a known population. The sample size was calculated as 339 with a 95% confidence interval. In our study, 404 anesthesiology and reanimation assistants participated voluntarily.

$$\eqalign{ n & = {{N{t^2}pq} \over {{d^2}\left( {N - 1} \right)\, + \,{t^2}pq}} \cr & = {{\left( {2842} \right) \cdot {{\left( {1,96} \right)}^2} \cdot \left( {0,5} \right) \cdot \left( {0,5} \right)} \over {{{\left( {0,05} \right)}^2} \cdot \left( {2841} \right) + {{\left( {1,96} \right)}^2} \cdot \left( {0,5} \right) \cdot \left( {0,5} \right)}} = 339 \cr}$$

N = Number of individuals in the universe.

n = Number of individuals to be sampled.

p = Frequency of occurrence of the event under investigation.

q= (1-p) Frequency of non-occurrence of the event under investigation.

d = Effect size.

t = The theoretical value in the t table at a certain degree of freedom and the determined error level.

The frequency (n) and percentage (%) are given for categorical variables. The relationships between categorical variables were analyzed with the chi-square test. In all analyses, *p* < 0.05 was considered to indicate statistical significance.

## Results

A total of 404 anesthesiology and reanimation assistants in Turkey participated in the study. Of the participants, 48.8% were male and 51.2% were female. A total of 65.8% of respondents had 3 or more years of experience, and 59.9% were working at a university hospital. A total of 87.9% of participants were aware of ERAS beforehand, and only 42.8% had undergone ERAS training. A total of 33.9% of the individuals who received this training did so through literature review. Cesarean delivery was performed at the institution of 93.8% of the participants. Of these participants, 48% stated that they applied ERAS protocols for cesarean delivery, and 33.4% stated that they had a team following the ERAS protocol. A total of 34.7% of this team consisted of physicians (Table 1).

**Table 1:** Questions 1–19 in the survey

**1- Sex**	**Female**			**Male**	
	207 (51.2)			197 (48.8)	
**2- How many years have you been an assistant?**	**1**	**2**	**3**	**4**	**5**
	69 (17.1)	69 (17.1)	95 (23.5)	76 (18.8)	95 (23.5)
**Years of education**	**< 2 yr**			**≥ 3 yr**	
	138 (34.2)			266 (65.8)	
**3- Which of the following is the institution you work for?**	**University Hospital**	**MHTR Hospital**	**Private University Hospital**	**City Hospital**	**Affiliate Hospital**
	242 (59.9)	105 (26.0)	12 (3.0)	38 (9.4)	7 (1.7)
**4- Have you ever heard of ERAS?**	**Yes**			**No**	
	355 (87.9)			49 (12.1)	
**5- Have you received training about ERAS?**	**Yes**			**No**	
	173 (42.8)			231 (57.2)	
**6- If your answer to the previous question is yes, where did you receive training about ERAS?**	**Online**	**Literature**		**Congress**	**Society**
	107 (26.5)	137 (33.9)		58 (14.3)	26 (6.5)
**7- Do you think ERAS protocols are helpful?**	**Yes**			**No**	
	371 (91.8)			33 (8.2)	
**8- Does your institution perform cesarean delivery?**	**Yes**			**No**	
	379 (93.8)			25 (6.2)	
**9- Do you apply ERAS protocols in cesarean delivery?**	**Yes**			**No**	
	194 (48.02)			210 (51.98)	
**10- Do you have a team that monitors the implementation of ERAS protocols?**	**Yes**			**No**	
	135 (33.4)			269 (66.6)	
**11- If your answer to the previous question is yes**,** who makes up this team?**	**Doctor (Assistant + Specialist)**		**Nurse**		**Other (physiotherapist**,** dietitian…)**
	140 (34.7)		44 (10.9)		34 (8.4)
**12- Do you inform your patients about the procedures before**,** during and after cesarean delivery?**	**Yes**		**No**		**Sometimes**
	323 (79.9)		80 (19.8)		1 (0,25)
**13- Do you administer antiacids and histamine H2 receptor antagonists as premedication to reduce the risk of aspiration pneumonia?**	**Yes**		**No**		**Sometimes**
	252 (62.4)		146 (36.1)		6 (1.5)
**14- Do you recommend bowel preparation before cesarean delivery?**	**Yes**		**No**		**Sometimes**
	145 (34.9)		257 (63.6)		2 (0.5)
**15- Do you use preoperative sedation for a planned cesarean delivery?**	**Yes**		**No**		**Sometimes**
	77 (19.1)		325 (80.4)		2 (0,5)
**16- Do you recommend drinking clear liquids (such as fruit juice without particles or coffee or tea without milk) up to 2 h before cesarean delivery?**	**Yes**		**No**		**Sometimes**
	178 (44.1)		223 (55.2)		3 (0,7)
**17- Do you recommend light nutrition up to 6 h before cesarean delivery?**	**Yes**			**No**	
	280 (69.3)			124 (30.7)	
**18- Do you recommend nondiabetic patients to drink oral carbohydrate liquid 2 h before cesarean delivery?**	**Yes**		**No**		**Sometimes**
	120 (29.8)		283 (70.0)		1 (0.2)
**19- Do you recommend optimal pregnancy weight gain management because maternal obesity (BMI > 40) increases the risks of maternal and fetal complications?**	**Yes**			**No**	
	278 (68.8)			126 (31.2)	

MHTR: Ministry of Health Training and Research, H2: Histamin 2, BMI: Body Mass Index, Values are expressed as the number of people (%)

A total of 79.9% of the participants provided information regarding the procedures before, during, and after the cesarean delivery, and 62.4% administered antiacids and histamin-2 (H2) receptor antagonists as premedication to mitigate the risk of aspiration pneumonia. A total of 34.9% of repondents recommended bowel preparation before cesarean delivery. A total of 80.4% of respondents did not use preoperative sedation for planned cesarean delivery. A total of 55.2% of respondents did not recommend consuming clear liquids, such as juice without pulp or nondairy coffee or tea, within 2 h prior to a cesarean delivery. It was recommended by 69.3% of respondents to follow a light diet for up to 6 h before a cesarean delivery. 70% of participants did not suggest the consumption of oral carbohydrate fluids by nondiabetic patients two hours prior to a cesarean delivery. A total of 68.8% of respondents recommended managing pregnancy weight gain to optimal levels due to the increased risk of maternal and fetal complications associated with maternal obesity (Table 1).

Additionally, 84.2% of respondents recommended controlling blood pressure to reduce the incidence of maternal and fetal morbidity during cesarean delivery among patients with chronic hypertension. Furthermore, 82.5% of respondents recommended regular monitoring of glucose levels due to the increased incidence of maternal and fetal morbidity associated with gestational diabetes mellitus. Since anemia during pregnancy is associated with low birth weight, preterm delivery, and increased perioperative morbidity and mortality rates, 81.4% of the participants aimed to identify and correct anemia. A total of 29.9% of the participants had established a protocol for correcting anemia. A total of 91.6% of the participants advised cessation of maternal smoking during pregnancy and the preoperative period to prevent mortality and morbidity (Table 2).

**Table 2:** Questions 20–32 in the survey

**20- Do you recommend blood pressure control because chronic hypertension increases the incidence of maternal and fetal morbidity and cesarean delivery?**	**Yes**				**No**				
	340 (84.2)				64 (15.8)				
**21-Do you recommend regular sugar monitoring because gestational diabetes mellitus increases maternal and fetal morbidity?**	**Yes**			**No**			**Sometimes**		
	333 (82.5)			70 (17.3)			1 (0.25)		
**22- Since maternal anemia during pregnancy is associated with low birth weight**,** preterm birth and increases perioperative morbidity and mortality rates**,** do you aim to identify and correct preoperative anemia?**	**Yes**				**No**				
	329 (81.4)				75 (18.6)				
**23-Do you have a protocol to correct anemia?**	**Yes**				**No**				
	121 829.9)				283 (70.1)				
**In those that exist**:	**Iron Replacement**				**Hematology (Internal Medicine) Consultation**				
	12 (9.9)				3 (2.4)				
**24- Do you recommend that the mother stop smoking during pregnancy and the preoperative period because it may cause mortality and morbidity?**	**Yes**				**No**				
	370 (91.6)				34 (8.4)				
**25- Do you recommend routinely administering a first generation cephalosporin to all women 60 min before the skin incision in cesarean delivery and adding azithromycin to women in labor or with ruptured membranes?**	**Yes**				**No**				
	229 (56.7)				175 (43.3)				
**26- What is your preferred anesthesia for cesarean delivery?**	**General Anesthesia**				**Regional Anesthesia**				
	14 (3.5)				390 (96.5)				
**27- If your answer is regional**,** which one do you prefer more?**	**Spinal**			**Epidural**			**Combined**		
	328 (82.4)			13 (3.2)			49 (12.4)		
**28- If your answer is regional anesthesia**,** which one do you apply to prevent hypotension due to regional anesthesia?**	**Crystalloid**	**Colloid**		**Ephedrine**		**Phenylephrine**		**Ephedrine + Crystalloid combination**	
	330 (81.7)	58(14.4)		333 (82.4)		22 (5.4)		235 (58.1)	
**29 - Hypothermia during cesarean delivery: what precautions do you take to prevent hypothermia due to surgical site infection**,** myocardial ischemia**,** change in drug metabolism**,** coagulopathy**,** prolongation of hospitalization**,** shivering**,** and decreased skin integrity?**	**Air Heating**		**Intravenous Fluid Warming**		**Increasing the Operating Room Temperature**			**None**	
	272 (67.3)		119 (29.5)		183 (45.3)			55 (13.6)	
**30- Do you pay attention to euvolemia by calculating perioperative and intraoperative fluid?**	**Yes**			**No**			**Sometimes**		
	356 (88.2)			45 (11.1)			3 (0.7)		
**31- Do you recommend chewing gum to your patients if delayed oral intake is planned?**	**Yes**				**No**				
	71 (17.6)				333 (82.4)				
**32- Do you recommend fluid preload and lower extremity compression to reduce the incidence of intraoperative and postoperative nausea and vomiting and hypotension?**	**Yes**				**No**				
	300 (74.3)				104 (25.7)				

Values are expressed as the number of people (%)

A total of 56.7% of participants suggested the routine use of a first-generation cephalosporin for all women who underwent cesarean delivery at least 60 min before the incision on the skin, with the inclusion of azithromycin for those in labor or suffering from membrane rupture. Regional anesthesia was preferred by 96.5% of the participants, and 82.4% of them preferred spinal anesthesia. To mitigate hypotension resulting from regional anesthesia, 82.4% of patients utilized ephedrine. The combination of ephedrine and crystalloid was the most preferred, with a rate of 58.1%. To prevent hypothermia during a cesarean delivery, 67.3% of the participants utilized the air warming method. Additionally, in 88.2% of the participants, euvolemia was confirmed by calculating fluid levels during the perioperative and intraoperative periods. A total of 17.6% of participants recommended chewing gum in cases of delayed oral intake, and 74.3% of respondents suggested fluid preloading and compression of the lower extremities to minimize the occurrence of nausea, vomiting, and hypotension during and after surgery (Table 2).

To prevent nausea and vomiting during a cesarean delivery, 82.5% of patients utilized antiemetics. The most frequently administered monotherapy antiemetic agent was the 5-Hidroksitriptamin3 (5-HT3) antagonist (granisetron or ondansetron) at 35.1%. The combination therapy, which consisted of an antagonist of the 5-HT3 receptor and dexamethasone, was utilized by 19% of the respondents. Of the participants, 49.3% recommended feeding within 2 h of cesarean delivery, and 57.2% performed perioperative blood glucose monitoring for cesarean delivery. To minimize the risk of thromboembolism, 78.5% of respondents favored early mobilization techniques, and 40.3% recommended the combination of embolic stockings and early mobilization. A total of 49.3% of participants recommended prompt catheter removal following cesarean delivery. In terms of postoperative pain management, 87.2% of respondents suggested using paracetamol. A total of 75.2% of the participants received multimodal analgesia. The most prevalent multimodal analgesic therapy involved the use of nonsteroid anti-inflammatory drugs (NSAID) and paracetamol, with a frequency of 42.3%. A total of 94.8% of participants were concerned about protecting the newborn from hypothermia. Additionally, 76% of respondents routinely recommended neonatal airway and gastric aspiration, and 96.1% were concerned about the availability of a neonatal resuscitation team and equipment in all settings where cesarean delivery was performed (Table 3).

**Table 3:** Questions 33–42 in the survey

**33- Do you use antiemetics to prevent nausea and vomiting during cesarean delivery?**	**Yes**			**No**		**Sometimes**	
	333 (82.5)			70 (17.3)		1 (0.25)	
**34- If your answer to the previous question is yes**,** which one do you use?**	**Dexamethasone**	**5HT3 antagonist**			**Monotherapy: 5HT3 antagonist**		**Combined therapy: 5HT3 antagonist**,** Dexamethasone**
	147 (36.4)	290 (71.8)			142 (35.1)		77 (19.0)
**35- Do you recommend feeding within 2 h after cesarean delivery?**	**Yes**				**No**		
	199 (49.3)				205 (50.7)		
**36- Do you check perioperative blood sugar during cesarean delivery?**	**Yes**			**No**		**Sometimes**	
	237 (57.2)			166 (41.1)		7 (1.7)	
**37- How do you take precautions for thromboembolism risk?**	**Embolism Stocking**	**Heparin**			**Early Mobilization**		**Embolism stocking**,** Heparin**
	272 (67.3)	82 (20.3)			317 (78.5)		163 (40.3)
**38- Do you recommend immediate removal of the catheter after cesarean delivery?**	**Yes**			**No**		**Sometimes**	
	199 (49.3)			204 (50.5)		1 (0.2)	
**39- What do you use for postoperative analgesia?**	**Paracetamol**	**NSAID**			**Opioids**		**Regional anesthesia**
	352 (87.2)	189 (46.8)			155 (38.4)		216 (53.5)
**Analgesic selection**:	**Multimodal analgesia**	**Monotherapy Paracetamol**			**Paracetamol**,** NSAID**		**Paracetamol**,** Regional anesthesia**
	304 (75.2)	61 (15.0)			171 (42.3)		65 (16.0)
**40- Are you careful to protect the newborn from hypothermia?**	**Yes**				**No**		
	383 (94.8)				21 (5.2)		
**41- Do you routinely recommend newborn airway and gastric aspiration?**	**Yes**			**No**		**Sometimes**	
	307 (76.0)			96 (23.8)		1 (0.2)	
**42- Do you pay attention to the presence of neonatal resuscitation teams and equipment in all settings where cesarean delivery are performed?**	**Yes**				**No**		
	388 (96.1)				16 (3.9)		

5-HT3: 5-Hidroksiriptamin3, NSAID: Nonsteroid anti-inflammatory drugs, Values are expressed as the number of people (%)

A total of 95.5% of those with 3 years or more of education had heard of ERAS. There was a statistically significant correlation between the number of years of specialty training and the number of participants who had previously heard of ERAS (*p* < 0.001). A total of 49.2% of those with 3 years or more of education had received training in ERAS. There was a statistically significant relationship between years of education and assistants’ training in ERAS (*p* < 0.001) (Table 4).

**Table 4:** Comparison of the answers to the question based on years of education with “Have you ever heard of ERAS?” And “Have you received training on ERAS?”

	Years of education		
Have you ever heard of ERAS?	≤ 2 years *n*(%)	3 years ≤ *n*(%)	*p*
Yes	101(73,20)	254(95,5)	**< 0**,001*
No	37(26,8)	12(4,5)	
**Have you received training on ERAS?**			
Yes	42(30,4)	131(49,2)	**< 0**,001*
No	96(69,6)	135(50,8)	

Values are expressed as the number of people (%), *p < 0.05 chi-square test

Of those who implemented ERAS protocols during cesarean delivery procedures, 71.6% were employed at a university hospital. There was a statistically significant difference between the application of ERAS protocols in cesarean delivery operations and the institution where the assistants worked (*p* < 0.001) (Table 5).

**Table 5:** Application status of ERAS protocols in cesarean section surgeries according to hospital type

	Do you apply ERAS protocols in cesarean section surgeries?		
Which of the following institutions do you work for?	Yes *n*(%)	No *n*(%)	*p*
Affiliate Hospital	3(1,50)	4(1,90)	**< 0**,001*
Private University Hospital	6(3,10)	6(2,90)	
Ministry of Health Training and Research Hospital	35(18,0)	70(33,30)	
City Hospital	11(5,70)	27(12,9)	
University Hospital	139(71,6)	103(49,0)	

Values are expressed as the number of people (%), *p < 0.05 chi-square test

## Discussion

Participants were predominantly from university hospitals and had more than three years of training. Although awareness of ERAS was high, the level of training in this area was lower than expected. Most participants believed in the benefits of ERAS, but very few followed ERAS recommendations for cesarean delivery. Most of the participating institutions did not have a team following ERAS protocols, and if they did, the team consisted primarily of surgeons and anesthesiologists. Fewer participants recommended clear fluids up to two hours before surgery and oral carbohydrate solutions up to two hours before cesarean delivery for nondiabetic patients than ERAS recommendations in the preoperative period for cesarean delivery. Most participants were implementing all intraoperative ERAS recommendations. Adherence to postoperative recommendations was also high, but few participants recommended the use of postoperative chewing gum if oral intake was planned to be delayed, early feeding within two hours of delivery, and early catheter removal.

Sedation can delay skin-to-skin contact between mothers and babies and delay breastfeeding. Therefore, due to its potentially harmful effects on the mothers and newborns, sedation before cesarean delivery is not recommended in ERAS protocols [2]. The high rate of acceptance of this recommendation indicates that participants were careful to consider the impact on the newborn before using any medication.

Although aspiration pneumonia is a rare condition, it remains a significant cause of maternal mortality during cesarean birth [8]. The high rate of antacid and H2-receptor antagonist use by participants indicates an awareness of the high risk of aspiration in pregnant patients.

A randomized controlled study of 130 patients by Lurie et al. revealed no benefit of bowel preparation prior to cesarean delivery [9]. We would have expected a greater percentage of participants not to recommend bowel preparation prior to cesarean delivery, but this finding suggests that a population is still clinging to old practices.

A study examined the gastric residual volume using ultrasound in 46 term pregnant women after 6 h of fasting. No visible solid food was detected in the gastric antrum, but in 17 patients, a gastric residual volume greater than 1.5 ml/kg was found, potentially increasing the risk of aspiration [10]. The lower-than-expected recommendation of 2 h of clear fluid intake preoperatively indicates that residents continue to be concerned about the risk of aspiration.

Patients who undergo delayed and rescheduled surgeries often leave the surgery hungry for 12 h or longer. The metabolic response to prolonged starvation leads to an enhanced organic response, manifested as increased insulin resistance and loss of muscle mass due to trauma [11]. Preoperative carbohydrate drinks have been shown to significantly improve patient comfort, especially in terms of hunger, thirst, fatigue, anxiety and nausea, by reducing postoperative insulin resistance [12]. We believe that the participants did not recommend oral carbohydrate fluids to nondiabetic patients 2 h before surgery because of fear of aspiration risk or difficulty with blood glucose regulation.

Smith and colleagues indicated that maternal anemia during pregnancy is a potentially reversible common risk factor associated with maternal and perinatal morbidity and mortality in the peripartum period [13]. Very few of the participants declared that they had a protocol for anemia. We believe that a multidisciplinary protocol should be created for the preoperative detection and treatment of anemia, and awareness should be raised on this issue.

A study indicated that adding azithromycin to antimicrobial prophylaxis in pregnant women with ruptured membranes can reduce the rate of maternal infections [14]. We believe that the addition of preoperative azithromycin in patients with membrane ruptures has been made known to many participants through our survey.

In elective cesarean surgery patients with spinal anesthesia, the time to initial postoperative analgesia requirement is longer and the 1-minute Apgar score is greater than that in patients with general anesthesia [15]. It has been observed that general anesthesia has negative effects on maternal adaptation, comfort, and the health status of the newborn during the postoperative period of cesarean births [16]. We believe that participants prefer regional anesthesia especially spinal anesthesia because it was easier and faster to administer.

It is assumed that chewing gum stimulates early recovery of gastrointestinal function through cephalo-vagal stimulation, reducing postoperative ileus [17]. In a randomized controlled trial involving 3,149 women who underwent cesarean delivery, chewing gum was administered for 3–6 sessions immediately after and up to 12 h postoperatively, resulting in early recovery of bowel function during the postoperative period [18]. The fact that participants did not recommend chewing gum may be due to a preference for early scheduling of oral intake and lack of adoption by participants due to weak evidence in guidelines or lack of knowledge on the topic.

Due to the complex pathophysiology of nausea and vomiting, a multimodal approach consisting of a combination of different antiemetic agents has been shown to be the most effective method for preventing intraoperative and postoperative nausea and vomiting in patients undergoing cesarean delivery [19]. Most participants used monotherapy, and few used combination therapy. The most preferred combination therapy was an 5-HT3 antagonist in conjunction with dexamethasone.

In a meta-analysis involving 1,911 patients, it was established that early oral intake after cesarean delivery under regional anesthesia not only accelerates the return of bowel function and surgical recovery but also reduces gastrointestinal complications [20]. Most of the participants believed that feeding may need to be delayed for 2 h, which is a traditional routine practice after anesthesia.

The surgical stress response leads to the release of catabolic hormones and the inhibition of insulin function, resulting in the development of hyperglycemia [21]. Since the majority of cesarean delivery patients were young and had fewer comorbidities, the participants may have thought that blood glucose was not monitored as strictly as expected.

A study comparing the time before and after the use of pneumatic compression stockings to prevent TE after cesarean delivery showed a significant reduction in pulmonary embolism deaths [22]. Early mobilization, theoretically, can improve postoperative outcomes, including the rapid return of bowel function, reduced risk of thrombosis, and shorter lengths of hospital stay [23]. Early mobilization and the use of embolic stockings to prevent TE were adopted by many participants in our survey.

The evaluation of published randomized clinical trials regarding the timing of urinary catheter removal after elective cesarean delivery has shown that early removal of the urinary catheter is associated with a significant reduction in the incidence of dysuria, urinary frequency, and bacteriuria [24]. The low proportion of participants who recommended immediate removal of the urinary catheter after cesarean delivery may be due to the belief that monitoring urine output may reduce urinary tract injuries and prevent postoperative urinary retention.

Postoperative pain is associated with prolonged hospital stays, surgical complications, chronic pain, and delays early mobilization. In one study, 60 patients who were scheduled for elective cesarean delivery under spinal anesthesia were divided into two groups, those who received a bilateral erector spinae plane (ESP) block and those who received a thoracoabdominal plane (TAP) block at the end of the surgery. The ESP block was found to provide more effective and prolonged analgesia than the TAP block and was associated with less tramadol consumption [25]. Most of the participants were using multimodal analgesia. The most frequently utilized analgesic combination was a combination of non-steroidal anti-inflammatory drugs (NSAIDs) and paracetamol, while the most commonly used monotherapy was paracetamol. Inadequate pain management after cesarean delivery delays breastfeeding, mother-newborn skin-to-skin contact, and mobilization, reducing patient conformity. Recently, with increased access to ultrasound, the administration rates of plan blocks for multimodal analgesia have progressively increased.

Cautions are recommended to protect newborns from hypothermia, such as maintaining the appropriate temperature in operating and delivery rooms, drying the newborn, and using prewarmed blankets, radiant warmers, or incubators [26]. The majority of our participants knew that newborns are susceptible to hypothermia due to their high surface area-to-volume ratio and low amount of brown adipose tissue, so they recommended taking precautions.

A vigorous, crying newborn, even with meconium in the amniotic fluid, does not routinely require aspiration and tactile stimulation. This is because unnecessary airway aspiration can lead to bradycardia. However, aspiration should be performed in the presence of secretions causing airway obstruction or in patients requiring positive pressure ventilation [27]. Most participants still recommended routine neonatal airway and gastric suctioning, and we believe that education in this area should be increased.

The limitations of this study include the fact that it was conducted only on anesthesia assistants and that the results may not be generalizable to other healthcare professions. The difficulty in reaching participants had a significant impact on the study.

## Conclusions

The majority of the participants in our survey were anesthesia assistants employed at the university hospital. They were generally aware of the usefulness of the ERAS recommendations. Nevertheless, some preoperative and postoperative recommendations were implemented by fewer participants. Although awareness of ERAS is high, we found that ERAS implementation rates are low due to a lack of multidisciplinary team formation and inadequate training.

## Data Availability

The data that support the findings of this study are not openly available due to reasons of sensitivity and are available from the corresponding author upon reasonable request.

## Abbreviations

ERAS: Enhanced recovery after surgery
OECD: Organization for Economic Cooperation and Development
CDG: Cesarean Delivery Guide
TE: Thromboembolism
SPSS: Statistical Package for the Social Sciences
MHTR: Ministry of Health Training and Research
H2: Histamin2
BMI: Body Mass Index
5-HT3: 5-Hidroksitriptamin3
NSAID: Nonsteroid anti-inflammatory drugs
ESP: Erector spinae plane
TAP: Thoracoabdominal plane
